# How hypoxia-induced diabetes develops and is maintained in children born preterm

**DOI:** 10.3389/fendo.2025.1720875

**Published:** 2026-01-26

**Authors:** Eung-Kwon Pae, Ronald M. Harper

**Affiliations:** 1 School of Dentistry, University of Maryland, Baltimore, MD, United States; 2 Department of Neurobiology, University of California at Los Angeles, Los Angeles, CA, United States

**Keywords:** beta-cells, insulin, KCC2, NKCC1, sympathetic outflow, WNK1

## Abstract

A puzzling metabolic question is the emergence of rapid-onset diabetes in the postnatal period of pre-term infants without the usual preceding prodromal characteristics. The etio-pathophysiology is unclear, but continues to be a concern, since the prevalence remains a significant health issue. We hypothesize that this new diabetes type is hypoxemia-driven from compromised ventilation *via* periodic breathing or apnea of infancy during the immediate postnatal period. The resulting intermittent hypoxia leads to elevated cytosolic chloride levels in pancreatic beta-cells affecting insulin secretion and disturbed glucose transporter (GLUT) 4 function resulting from lowered With-no-lysine (k) kinase (WNK)1 levels in the skeletal musculature. In addition, the peripheral cellular effects are coupled with prolonged elevated sympathetic outflow elicited by the disrupted breathing. This mini-review discusses current research gaps and provides insights into potential interventions for the widespread epidemic of Type 1 and Type 2 diabetes.

## 1 Introduction

Intracellular mechanisms regulating insulin secretion in pancreatic beta-cells use redundant reciprocal control processes to maintain optimal insulin levels for survival. The human body cannot afford reduced accuracy in insulin control leading to rapid downregulation of glucose levels, even for short time periods. However, homeostatic control of high blood glucose levels from insufficient insulin is better tolerated. Of the many redundant insulin control mechanisms regulating high glucose levels, lowered intracellular chloride ion levels induced by intermittent hypoxic conditions (IH), is commonly seen in critically ill COVID-19 patients (1) or rodent models for premature infants experiencing periodic breathing (2). Low chloride levels in pancreatic beta-cells down-regulate insulin secretion. Downregulation of phosphorylated with-no-lysine kinase1 (pWNK1) levels in skeletal muscle lowers glucose transporter 4 (GLUT4) on the muscle cell surface and escalates insulin resistance (3) as commonly found in Type 2 diabetic patients. In this review, we offer an integrative hypothesis using first, a *de novo* proposal based on a ubiquitous WNK1 pathway in pancreatic beta-cells and neuronal cells if IH continues; second, a proposal based on a disinhibitory outcome of γ-aminobutyric acid (GABA)-GABAergic receptor coupling inside GABAergic neuronal cells. This excitotoxic disinhibition and oxidative stress-induced apoptosis due to IH will force GABAergic neurons to undergo apoptotic events. Elevation of sympathetic nerve tone relative to parasympathetic outflow from periodic breathing or IH exposure results in higher levels of circulating norepinephrine (4). If IH continues, reflexive sympathetic circuitry can trigger higher stress hormone outflow into the blood stream. We suggest that triggering hypoxia-chloride processes, followed by elevated corticosteroid hormone levels processed by hypothalamic neural circuitry underlie glucose dysregulation in infants who undergo IH exposure with sustained periodic breathing.

### 1.1 Intermittent hypoxemia reduces cytosolic chloride ion levels in pancreatic beta-cells

When pancreatic beta-cells are exposed to IH for 1h, cytosolic chloride ion levels are downregulated (2). Such short-term IH exposure *in vivo* does not reduce beta-cell counts (5); therefore, to examine processes affecting insulin release, we focused investigations on ion-transporters. We observed that IH exposure down-regulates intracellular chloride levels in beta-cells as NKCC1 (potassium chloride co-transporter or chloride importer) levels decrease and KCC2 (chloride exporter) levels increase. We reported that insulin secretion declined under IH conditions, yet increased significantly after being treated with a KCC2 inhibitor, even under IH exposure. This set of observations suggests that controlling intracellular chloride levels in beta-cells may be critical for insulin secretion.

Inhibition of the K^+^-Cl^–^ co-transporter 2 (Kcc2), a potassium chloride co-transporter in clonal rodent beta-cells, increases basal and glucose-stimulated insulin secretion and Ca^2+^ uptake (6). We found that this phenomenon appeared in conjunction with an increased density of Kcc2, a potassium chloride co-transporter exporter, and low chloride in the cytosol as well as plasma membrane of primary rat pancreatic beta-cells after transient IH conditioning (2). It appears, first; as chloride ions are extruded, potassium ions efflux as well (7), second; normally, membrane depolarization would not occur due to leaking potassium ions from the beta-cells, then, third; insulin secretion levels decline due to reduced Ca^2+^ influx.

### 1.2 IH disrupts autonomic nervous system activity, in particular, elevating sympathetic tone in central neuronal circuits

Shortly after birth, a slight predominance of sympathetic tone appears, followed by a parasympathetic dominance in normal term-birth infants (8). However, preterm infants, particularly those experiencing IH exposure through periodic breathing or apnea of infancy, show elevated sympathetic tone (9, 10). Part of the sympathetic tone exacerbation may result from chronic mild stress on the GABAergic system in the paraventricular nucleus (PVN) of the hypothalamus (11) due to brief IH. The PVN contains neurons that project directly to sympathetic preganglionic nuclei in the spinal cord (12); and is a major source of excitation to spinal sympathetic outflow. When PVN pre-sympathetic neurons are sufficiently impaired to reduce pre-sympathetic GABA release or if GABAergic neurons are lost, that altered outflow will play a critical role in regulating sympathetic responses to IH exposure (13), a sequence that may modify insulin secretion (14–16).

Elevated sympathetic tone would be further sustained if the IH accompanying disordered breathing patterns damages Purkinje neurons or fastigial nuclei (FN) in the cerebellum, as has been demonstrated in animal models (17, 18). Cerebellar circuitry plays an essential role in sympathetic regulation, with the FN a major waystation of processed signals from Purkinje cells mediating that outflow (19). The FN, undampened by injured Purkinje cells from IH exposure (12), will enhance sympathetic outflow, with consequent reduced heart rate variability (a characteristic of preterm cardiac patterns) (20), increased cortisol release, and disrupted insulin outflow. Dampening and recovery of blood pressure changes signaled by vestibular and solitary tract nuclei are modulated by circuitry to Purkinje cells and FN *via* climbing fibers (17, 21).

Afferent information processed in the arcuate nuclei of the hypothalamus (ARC) passes to the PVN (22). The ARC controls multiple physiological functions, being a central hub of metabolic regulation, including glucose homeostasis, blood pressure, and innate immune responses, in addition to feeding. Thus, this set of nuclei regulates energy intake and expenditure. Interestingly, ARC contains abundant GABAergic cells; yet, its noradrenergic innervation exerts an opposite effect to that of the PVN on several neuroendocrine regulation aspects (23). The PVN and ARC nuclei exhibit reciprocal roles in central noradrenergic regulation. As described above, the PVN is a critical site for central integration of sympathetic outflow. Most importantly, excitatory and inhibitory interactions within the PVN determine sympathetic tone depending on whether ‘inhibition or disinhibition’ conditions prevail (24).

### 1.3 Continuing IH exposure aggravates the hypothalamus-pituitary-adrenal axis, elevating adrenocorticotrophic hormone

ACTH regulation and insulin control processes are well studied, and the neural-endocrine interactions have been the topic of intense investigation (25, 26). Intermittent hypoxia elevates ACTH, which enhances cortisol release *via* the limbic system (27). If this process continues, insulin secretion increases chronically; however, this physiological sequence needs to negotiate processes in the beta-cells described above, as well as timing of the responses to hypoxic challenges. Thus, ACTH action on insulin secretion could be chronic or subacute rather than an immediate reaction to IH exposure in preterm neonates, and further discussion of which is beyond the scope of this review.

## 2 Controlling cytosolic chloride

Our, and other studies on cytosolic changes of chloride ion levels have been triggered by a historical series of investigations from the Sehlin lab (28), which indicate that chloride ions are non-passively distributed across the beta-cell plasma membrane, and that increased chloride permeability may partly mediate D-Glucose-induced depolarization of pancreatic beta-cells (28). However, the mechanical signaling cascade related to chloride-influx and membrane depolarization has only recently begun to be understood. The extent of the cytosolic chloride concentration needed to trigger the signaling cascade for insulin secretion is unclear. The answer is, in short, not much (29). Best and colleagues rekindled the chloride hypothesis by showing that beta-cells are equipped with a volume-sensitive anionic [Cl^–^] conductance (30), providing a functional link between hypotonic beta-cell swelling (31) and insulin secretion interpreted by beta-cell electrical activity (32).

### 2.1 Major apparatus controlling influx/efflux of chloride ions in beta-cells

To transport chloride ions across the plasma membrane, scores of Cl^−^ transporters and channels are available as an active or passive transfer apparatus, such as well-studied active Cl^−^ transporters: Na^+^-K^+^-2Cl^−^ cotransporter (NKCC), Na^+^-Cl^−^ transporter (NCC), Na^+^-driven Cl^−^/HCO3^−^ exchanger (NDCBE), and K^+^-Cl^−^ cotransporter (KCC) (33). NKCCs contribute to Cl^−^ uptake into cells using the electrochemical potential of Na^+^ generated by the Na^+^, K^+^-pump, while KCCs participate in Cl^−^ efflux from cells using the electrochemical potential of Na^+^ and K^+^ generated by the Na^+^, K^+^-pump. An increase in NKCC-mediated Cl^−^ influx elevates cytosolic chloride ion levels as NKCCs co-transporters are phosphorylated. The elevated total [Cl^−^]i induces an increase in the chemical potential of cytosolic Cl^−^, which elevates Cl^−^ efflux through Cl^−^ channels. The [Cl^−^]i increases until the elevation of Cl^−^ efflux through Cl^−^ channels equals the increase in Cl^−^ influx *via* NKCCs and the like; at this point, the chloride change reaches equilibrium. The opposite phenomenon occurs when NKCC-mediated Cl^−^ influx is reduced. As [Cl^−^]i declines, so does Cl^−^ efflux through Cl^−^ channels. Then, the reduction in [Cl^−^]i reaches equilibrium at a point where the decrease in Cl^−^ efflux through Cl^−^ channels or KCCs and similar processes equals the reduction of Cl^−^ influx *via* NKCCs. An interesting aspect regarding Cl^−^ flux is that changes in [Cl^−^]i due to Cl^−^ channel activity are transient, whereas alterations in Cl^−^ flux due to Cl^−^ transporter activity are persistent (34).

Recently, we introduced the role of chloride ions and their corresponding co-transporters to the forefront of the hypoxia discussion, because the canonical potassium channel system is relatively unaffected by hypoxic exposure (2). However, the density of potassium chloride co-transporters, a chloride exporter, on the plasma membrane responds sharply to IH exposure and lowers cytosolic levels of chloride markedly, which presumably underlies the significant reduction of insulin secretion during intermittent hypoxic conditions. Similarly, alterations in neuronal cells in a developing brain are induced by sustained IH exposure, and those changes should be viewed in the context of cytosolic chloride ion levels as Ben-Ari previously offered using neuronal cells (35).

### 2.2 Chloride control and pre-sympathetic drive in the PVN – a suggested ‘*de novo* hypothesis’

Earlier, Ben-Ari described how γ-aminobutyric acid (GABA), a major source of inhibition in the adult brain, could function as an excitatory neurotransmitter, leading to a negative shift in the reversal potential for chloride ions *via* delayed expression of a chloride exporter, KCC2, in the immature brain. Then, he suggested that developing neurons promoting growth and synaptic formation require the avoidance of potential toxic effects of a mismatch between GABA-mediated inhibition and glutamatergic excitation in neural conditions showing dual excitatory/inhibitory effects of GABA in developmental stages based on cytosolic chloride-ion levels (35).

**Figure 1:**
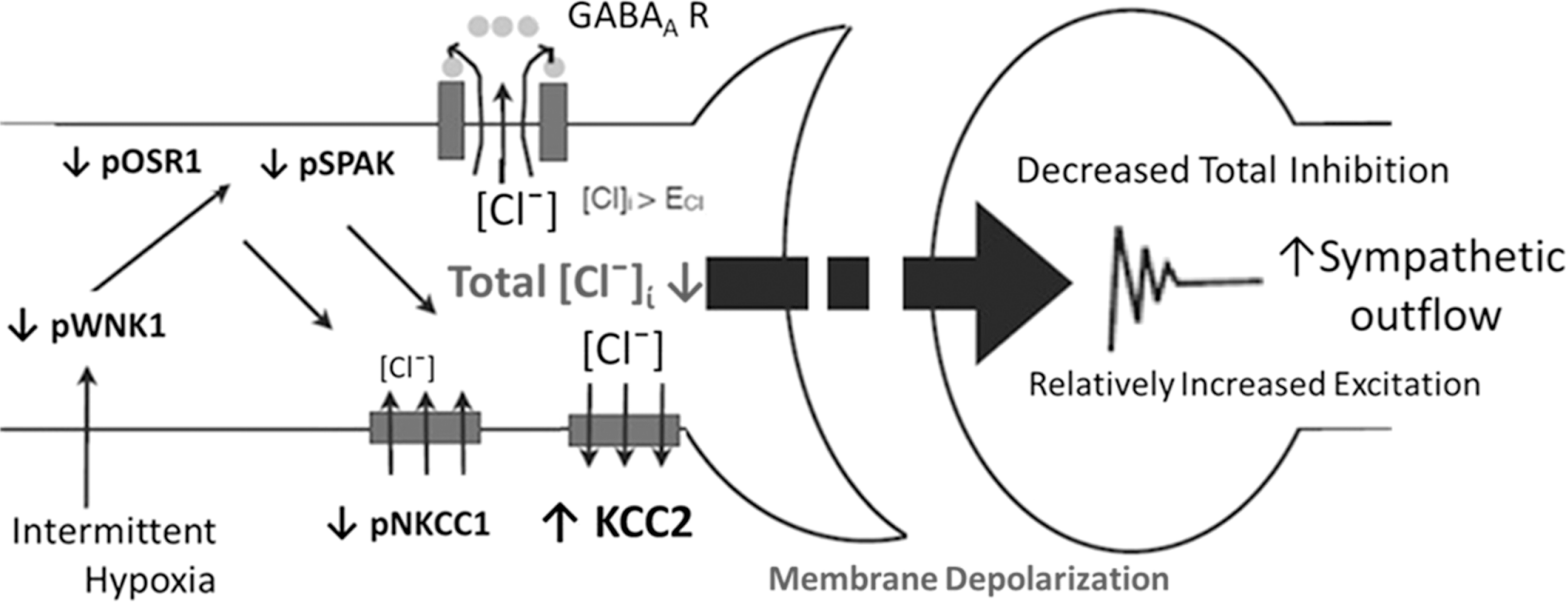
Cartoon summary of the expected sequence of events in injured neuronal cells of the FN (2) and PVN in response to brief IH. Once the level of phosphorylated WNK1 (or pWNK1) is down-regulated, and KCC2 levels are up-regulated by IH insults (as also shown in **Figure 2a**), cytosolic chloride levels should be maintained low; however, the total neural outcome of GABAergic neuronal cells in the IH-primed FN and PVN will tip toward excitation (yet, diminished total GABA inhibition), and followed by ‘autophosphorylation process’ as demonstrated in **Figure 2**. See below for more explanation on the WNK1-SPAK/OSR1-NKCC1/KCC2 signaling pathway balancing or unbalancing chloride levels in animal cells.

Recent studies found this an ‘attractive’ story to show a remarkable parallel to explain control of insulin release from pancreatic beta-cells based on chloride ion levels by the Di Fulvio lab and the Pae lab. In particular, we demonstrated that insulin secretion declined markedly in immature rodents treated under a brief IH condition; thus, the animals become diabetic shortly after exposure (2, 5). We have not confirmed yet whether the chloride transporter changes in accordance with functions occurring in GABAergic pre-sympathetic cells in hypothalamic nuclei, such as the PVN, and in the Fastigial Nuclei (FN) of the cerebellum (See **Figure 1**). With-no-lysine (k) kinase (WNK) is named for the feature that the molecule, a serine/threonine kinase lacking catalytic lysine in subdomain II, is crucial for binding to ATP. WNK1 is a chloride-sensing protein kinase ubiquitously expressed in tissues and cell lines in response to osmotic stress, the functional mechanisms of which were first analyzed by the M. Cobb Lab (36). Such changes include phosphorylation on NKCC1 or KCC2 transporters, along with changes in the WNK1 pathway, as conjectured by Watanabe and Fukuda (37).

## 3 How dephosphorylation occurs to OSR1 kinase, and what would occur if it does

**Figure 2:**
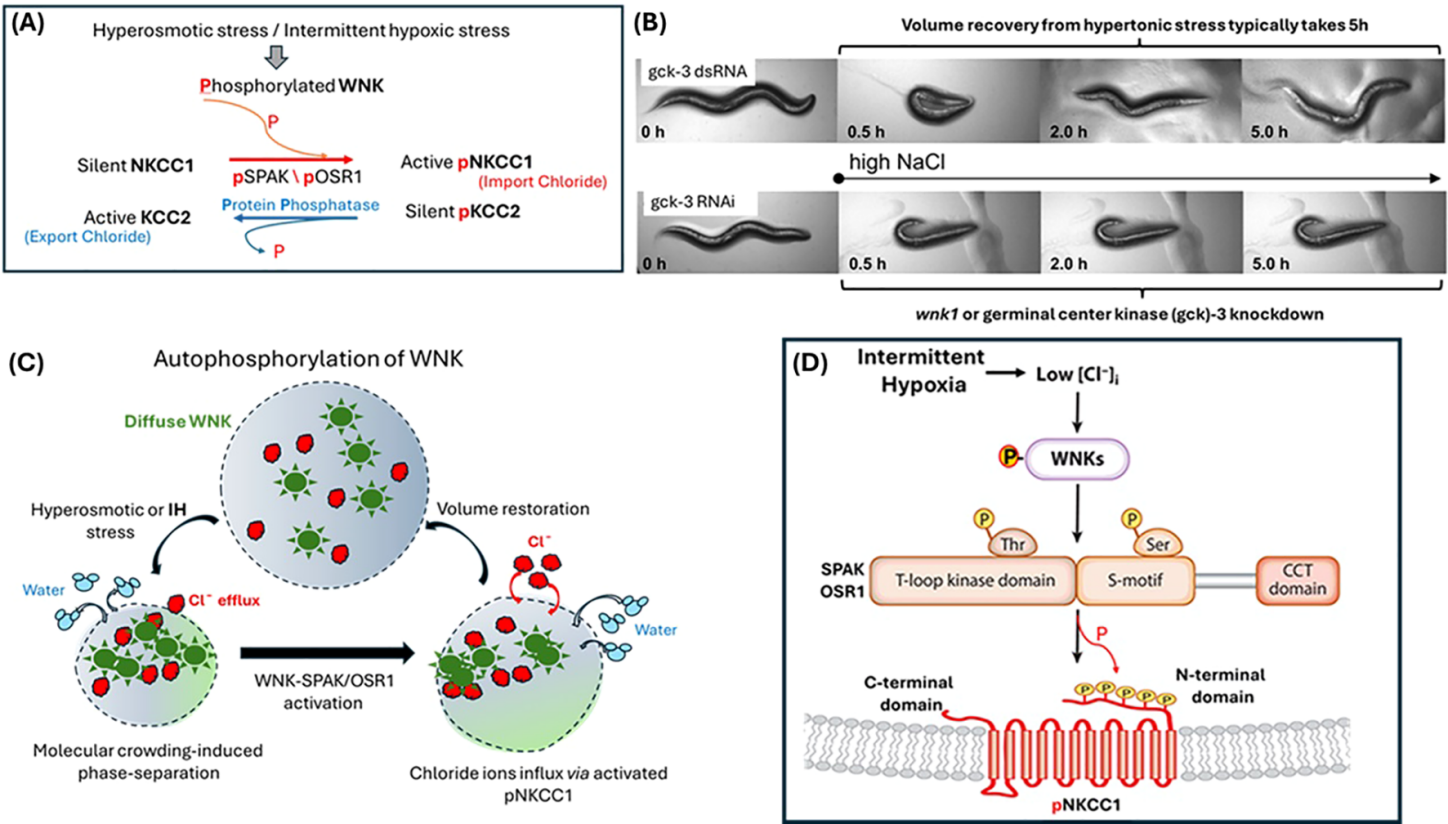
Autophosphorylation as a universal tactic to control cytosolic chloride levels for overcoming cell shrinkage in animals. **(a)** Regulation of cytosolic chloride levels by active phosphorylated WNK as a reciprocating toggle switch (41). **(b)** Systemic volume of C. Elegans is regulated by *wnk-1* for body locomotion, [Adapted from Choe and Strange] (42). RNA interference (RNAi) knockdown of the C. elegans GCK-VI kinase, GCK-3, inhibits total volume recovery from shrinkage as shown in the lower panel. Since WNK-1 activates GCK-3 *via* phosphorylation, silencing of either *wnk-1* or *gck-3* hampers volume recovery of the C. elegans. **(c)** The autophosphorylation process in mammalian cells to rescue cells from shrinkage is triggered by physical crowding of WNK molecules subsequent to chloride efflux (43). **(d)** At low chloride conditions, WNK molecules liberate chloride ions to phosphorylate down-stream molecules like SPAK and OSR1, and which in turn, phosphorylate [or activate, as shown in Panel **(a)**] NKCC1 chloride importers [Adapted from Goldsmith and Rodan] (7).

The WNK1 structure shows an autoinhibitory domain and an obligatory autophosphorylation site in the activation loop, which uses typical regulatory mechanisms as exhibited in **Figure 2**. Phosphorylation of the activation loop residue controls the activity of protein kinase. WNK regulates the activity of stress-related serine-threonine kinases, STE20 (sterile 20)/SPS1-related proline/alanine-rich kinase (SPAK), and oxidative stress-responsive kinase 1 (OSR1), which are downstream targets of WNK1.

The OSR1 gene was first sequenced and confirmed from human cancerous tissues, including the pancreas (38). The sequence of OSR1 was 39% identical to that of human SOK (Ste20/oxidant stress response kinase-1), known to be activated by oxidative stress; therefore, the sequence was coined “OSR1.” Numerous studies confirmed that OSR1 directly phosphorylates N-terminal regulatory regions of potassium-chloride-transporters, *e.g*. NKCC1, and phosphorylates WNK1. Devoid of phosphorylated WNK1, phosphorylated OSR1 alone does not activate (or phosphorylate) a chloride importer NKCC1 (39); thus, auto-phosphorylation by chloride ion levels does not occur without OSR1 being phosphorylated despite the phosphorylation of WNK1.

If oxidative stress directly affects SPAK/OSR1, that process could, in turn, regulate WNK activity. Yet, no direct evidence links IH challenges to dephosphorylation of OSR1 thus far. Alessi et al., using double/single knock-in (KI) embryonic stem cells where WNK1 does not activate SPAK and OSR1, showed that NKCC1 was not phosphorylated or activated under knock-in conditions (40). They also demonstrated that WNK1 activity was elevated in the KI cells, indicating that SPAK/OSR1 significantly influences WNK activity. In their protocol, SPAK KI cells lowered NKCC1 activity in low chloride medium by one-half, exhibiting a less phosphorylated SPAK. In contrast, under identical conditions, OSR1 KI cells lowered NKCC1 activity further toward the lowest level, showing completely dephosphorylated NKCC1. This outcome may indicate that NKCC1 activity is affected by pOSR1 more than by pSPAK in lower chloride buffer.

As the cytosolic chloride level declines, WNK1 transfers phosphates to downstream molecules SPAK/OSR1. As OSR1/SPAK are phosphorylated, NKCC1 molecules are phosphorylated to become active. However, if OSR1 is insufficiently phosphorylated, NKCC1 is not sufficiently phosphorylated regardless of SPAK phosphorylation, where insufficient chloride influx by less-active NKCC1 function opposing active KCC2 function results in diminished insulin secretion (2).

### 3.1 Mechanisms for chloride influx/efflux as a redundant tool for insulin regulation under IH conditions

Sympathetic overdrive contributing to the derangement of glucose metabolism is occasionally found in patients with type 2 diabetes (44). Collectively, the association between diabetes and sympathetic surges provides insights into the prevalence of acute diabetes reported with COVID-19, a condition characterized by sustained periods of intermittent hypoxia (27). SARS-CoV-2 infection-induced stress exposes the sympathetic nervous system to pro-inflammatory cytokines, which may further exaggerate sympathetic discharge.

When beta-cells are attacked by viruses and hypoxemia simultaneously, chloride efflux begins immediately, followed by reduced insulin secretion (45). As demonstrated in a macrophage model, both viral particles and hypoxia trigger chloride efflux and the WNK1 pathway (46). As chloride levels decline in beta-cells, autophosphorylation processes by WNK1 begin. While WNK1 levels remain low, the acute diabetic condition is maintained, irrespective of whether the patient has acute pancreatitis or not.

Apart from diabetes, the question of how cystic fibrosis patients with an abnormal transmembrane conductance regulator (CFTR) avoid severe illness from contraction of SARS-CoV2 may be answered by their innate capability of trapping chloride ions inside the cells that is the cause of CFTR (47). Another example is that circadian rhythms can be modulated by variability of the cytosolic chloride level in the suprachiasmatic nucleus along with the degree of phosphorylation of chloride co-transporters and OSR1 in the WNK1 signaling pathway (48). Likewise, any other means to elevate phosphorylation of OSR1 or down-regulate sympathetic tone would ameliorate the reduction in insulin levels. Although a few more steps are needed to confirm this possibility, this hypothetical pathophysiology of diabetes induced by IH breathing suggests that focusing on providing appropriate ventilatory support to affected patients before the HPA axis takes over long-term glucocorticoid control would be beneficial.

### 3.2 Neural mechanisms reinforced by chloride effects - an integrated hypothesis

Prenatal hypoxia challenges can alter central respiratory drive by disrupting the development of chloride co-transporters, KCC2 and NKCC1 in Sprague-Dawley rats *via* altering topographical changes in distributions of GABA/GABA_A_ and glycine receptors (49). Our lab and others previously reported outcome effects brought by prenatal IH challenges on the heart, cerebellum (17), skeleton (50) and skeletal muscles (51). Neural maladaptation explains how an early IH challenge after birth sustains the endocrine effects, often for prolonged durations. Typically, autonomic imbalance (exaggerated sympathetic activity over parasympathetic withdrawal, as described earlier), emerges as a causal contributor of IH-induced altered catecholaminergic nerve innervation in mouse atria (52). Previously, we and others (53, 54) quantified topographical changes in immunohistochemically-labeled tyrosine hydroxylase (TH, a sympathetic marker) and calcitonin gene-related peptide (CGRP, a common marker for nociceptive nerves), and demonstrated that a network of CGRP-IR axons densely innervate the mouse atria where large nerve bundle entry points and a regional concentration of CGRP-IR axons are present. These findings suggest a significant potential for IH to play a role on peripheral neural interactions with endocrine functions *via* autonomic nervous circuitry, leading to anatomic alterations during early life.

We believe that modulated insulin secretion levels *via* a neural axis is one such adaptation, not from a shortage of beta-cells in numbers or function, but by neural influences on the hypothalamic arcuate nucleus (ARC) in early postnatal ages when periodic breathing is present (55). We acknowledge that brain and pancreatic tissues exposed to IH immediately after birth are vulnerable to molecular adaptation. We hypothesize that postnatal IH insults can lead to decreased cytosolic chloride levels which play a critical role in both elevated sympathetic outflow and down-regulated insulin secretion. Although many unanswered questions remain on mechanisms of action, we also observed that IH interferes with effects of γ-aminobutyric acid (GABA) on insulin secretion in postnatal rodents (56), where cytosolic chloride levels were challenged.

## 4 Closing remarks

This short review poses a number of open questions. Adaptation processes to reduced oxygen levels on physiology can be exerted immediately on exposure after birth. Very rapid changes occur at molecular, cellular and tissue levels if hypoxic conditions continue at, or above a certain frequency or severity. Here, we describe plausible mechanisms and offer hypothetical answers to understand what processes likely underlie disturbances in glucose homeostasis to intermittent hypoxic challenges in early human life. The processes appear complex. We suggest that, in addition to the level of insulin secretion from expected values of pancreatic beta cells, the consequences of hypoxic and accompanying sympathetic discharge effects on other functional organ units, including brain structures, skeleton (*i.e*., bone loss from excessive sympathetic discharge), and musculature should be considered. The response of organs to intermittent hypoxia are not simple output changes, but trigger other reactions that ripple through several peripheral or central organs *via* neurotransmitters and ion-transporters until homeostasis is achieved. The pathogenesis of the increased risk for Type-1 and -2 diabetes in children born preterm is unclear (57); however, the sum of these physiological responses to intermittent hypoxic breathing in the early postnatal period can contribute to significant long-term repercussions, including diabetes mellitus.
